# Dynamics of cytotoxic T cell subsets during immunotherapy predicts outcome in acute myeloid leukemia

**DOI:** 10.18632/oncotarget.7210

**Published:** 2016-02-05

**Authors:** Frida Ewald Sander, Anna Rydström, Elin Bernson, Roberta Kiffin, Rebecca Riise, Johan Aurelius, Harald Anderson, Mats Brune, Robin Foà, Kristoffer Hellstrand, Fredrik B. Thorén, Anna Martner

**Affiliations:** ^1^ TIMM Laboratory, Sahlgrenska Cancer Center, University of Gothenburg, Gothenburg, Sweden; ^2^ Department of Hematology, University of Gothenburg, Gothenburg, Sweden; ^3^ Department of Cancer Epidemiology, University of Lund, Lund, Sweden; ^4^ Department of Cellular Biotechnologies and Hematology, Sapienza University of Rome, Rome, Italy

**Keywords:** acute myeloid leukemia, immunotherapy, cytotoxic T cells, antigen-specific T cells, Immunology and Microbiology Section, Immune response, Immunity

## Abstract

Preventing relapse after chemotherapy remains a challenge in acute myeloid leukemia (AML). Eighty-four non-transplanted AML patients in first complete remission received relapse-preventive immunotherapy with histamine dihydrochloride and low-dose interleukin-2 in an international phase IV trial (ClinicalTrials.gov; NCT01347996). Blood samples were drawn during cycles of immunotherapy and analyzed for CD8^+^ (cytotoxic) T cell phenotypes in blood. During the first cycle of therapy, a re-distribution of cytotoxic T cells was observed comprising a reduction of T effector memory cells and a concomitant increase of T effector cells. The dynamics of T cell subtypes during immunotherapy prognosticated relapse and survival, in particular among older patients and remained significantly predictive of clinical outcome after correction for potential confounders. Presence of CD8^+^ T cells with specificity for leukemia-associated antigens identified patients with low relapse risk. Our results point to novel aspects of T cell-mediated immunosurveillance in AML and provide conceivable biomarkers in relapse-preventive immunotherapy.

## INTRODUCTION

Patients diagnosed with acute myeloid leukemia (AML) receive induction chemotherapy aiming at attaining the microscopic disappearance of leukemic cells and the re-appearance of normal hematopoiesis (complete remission, CR). The post-remission phase includes consolidation chemotherapy with the goal of eradicating undetectable leukemic cells. However, relapse in CR is common, in particular in older patients (> 60 years old), and significantly explains why a minority of adult AML patients achieve long-term leukemia-free survival (LFS) [1, 2].

Relapse in the post-consolidation phase of AML in CR likely results from the expansion of leukemic cells that have escaped the initial rounds of chemotherapy. While details regarding the role of cellular immunity for the elimination of residual leukemic cells remain largely unknown, experience from the use of allogeneic transplantation for relapse prevention in AML strongly implies a role for grafted T cells [2-4]. In non-transplanted AML patients, the role of T cells for the surveillance of leukemic cells is less well documented, but the results of smaller studies suggest that prognosis is favorably impacted by the emergence of anti-leukemic T cells in bone marrow or blood [5] and by the presence of mRNAs encoding leukemia-associated antigens [6]. These results have inspired the design of immunotherapies aiming at achieving T cell-mediated elimination of AML cells e.g. by the adoptive transfer of engineered T cells [NCT01864902, 7], by promoting autologous T cell function by vaccination [8], by use of bispecific antibodies linking T cells to leukemic cells [NCT02152956, 9], by countering mechanisms of T cell suppression [NCT02532231, 9], and by the administration of T cell-activating cytokines [NCT01885897, 10].

CD8^+^ (cytotoxic) T cells differentiate from antigen-inexperienced naïve T cells (T_N_) into central memory (T_CM_), effector memory (T_EM_) and effector (T_eff_) cell populations [11]. These T cell subsets are distinguished by phenotype and by their localization: naïve T cells (CD45RA^+^CCR7^+^) and T_CM_ (CD45RO^+^CCR7^+^) are localized mainly in secondary lymphoid organs whereas T_EM_ (CD45RO^+^CCR7^−^) and T_eff_ (CD45RA^+^CCR7^−^) cells circulate through non-lymphoid tissues [12]. Studies using adoptive cell transfer for therapeutic purposes imply that T cells at early stages of differentiation are superiorly efficacious in cancer immunotherapy as these cells are capable of self-renewal and may continuously provide new T_eff_ cells [13]. For the present study, we assessed the distribution of CD8^+^ T cell subsets in the peripheral blood of AML patients undergoing immunotherapy for relapse prevention with histamine dihydrochloride and low-dose interleukin-2 (HDC/IL-2). Our results suggest that the dynamics of T cell phenotypes during immunotherapy heralds remission maintenance and survival in AML.

## RESULTS

### Dynamics of cytotoxic T cell subsets during immunotherapy

**Figure 1:**
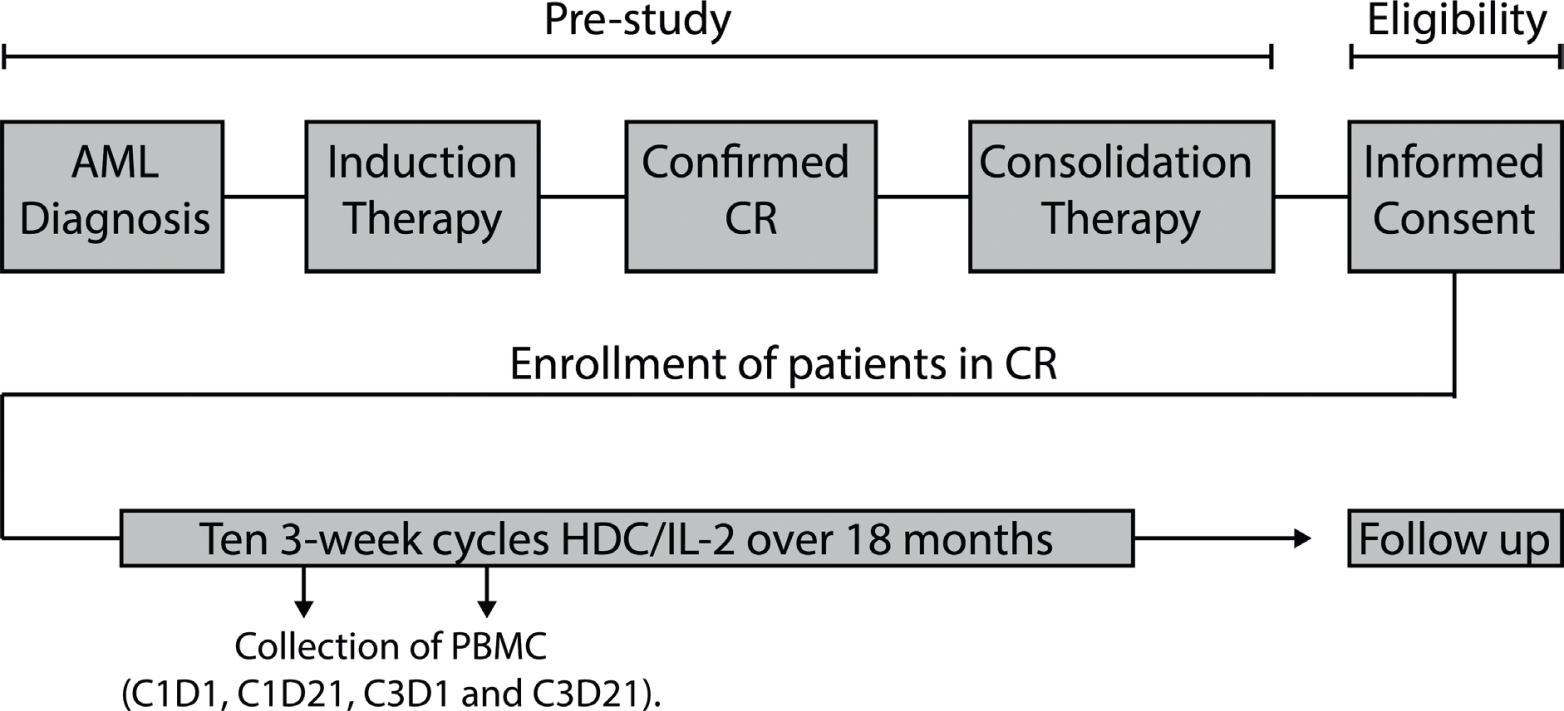
Overview of the Re:Mission phase IV trial Eligible AML patients in first complete remission (CR) received ten 3-week cycles of HDC/IL-2 over 18 months. Peripheral blood mononuclear cells (PBMC) were isolated from blood collected before and after cycles 1 and 3. Patients were followed-up for 6 months after completing the last treatment cycle.

**Figure 2:**
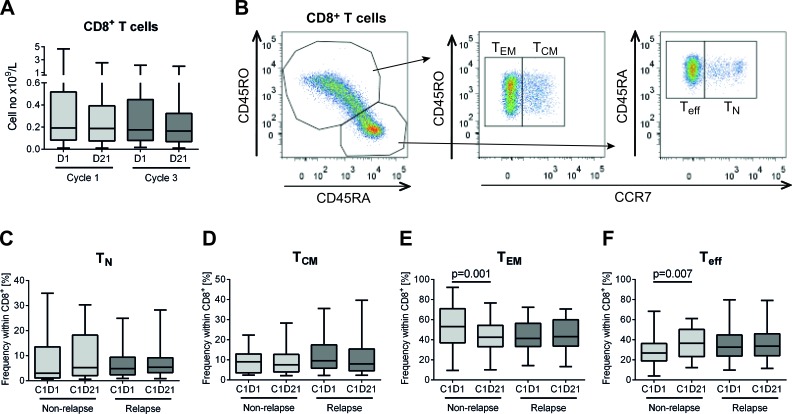
Distribution of CD8^+^ subsets in non-relapsing and relapsing AML patients during immunotherapy with HDC/IL-2 **A.** Blood counts of CD8^+^ T cells before (D1) and after (D21) the first and third cycles of HDC/IL-2 treatment (C1D1 *n* = 62; C1D21 *n* = 54; C3D1 *n* = 52; C3D21 *n* = 51). **B.** Gating strategy for determining naïve (T_N_; CD45RA^+^CCR7^+^), central memory (T_CM_; CD45RO^+^CCR7^+^), effector memory (T_EM_; CD45RO^+^CCR7^−^) and effector (T_eff_; CD45RA^+^CCR7^−^) cells within the CD8^+^ T cell compartment. C-F. Frequency of the CD8^+^ subpopulations T_N_ **C.**, T_CM_ **D.**, T_EM_ **E.** and T_eff_ **F.** cells in non-relapsing (*n* = 18) and relapsing (*n* = 26) patients at the onset (C1D1) or end of (C1D21) the first cycle of immunotherapy. Statistical analysis was performed by Student's paired *t*-test.

AML patients in first CR received 10 consecutive 3-week cycles of HDC/IL-2 in the post-consolidation phase, as outlined in Figure 1. Peripheral blood collected before and after the first and the third cycles of immunotherapy was analyzed for CD8^+^ T cell content and phenotype. During the treatment cycles, HDC/IL-2 did not alter the absolute counts of CD8^+^ T cells in blood (Figure 2A). Also, the CD8^+^ T cell counts (above or below the median) before or after therapy did not impact on relapse risk (data not shown).

**Figure 3:**
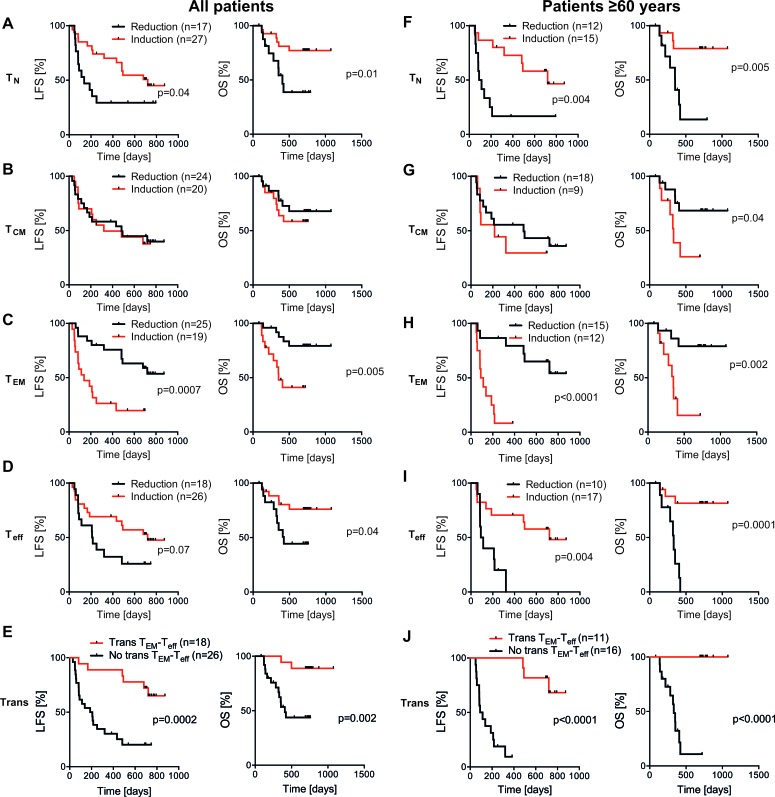
Impact of altered distribution of CD8^+^subsets on the clinical outcome of patients receiving HDC/IL-2 In **A**-**D.** all patients, and in **F**-**I.** patients ≥ 60 years old, were dichotomized based on induction or reduction of the frequency of CD8^+^ T cell subsets during the first treatment cycle, followed by analyses of LFS and OS by the logrank test. In **E.** all patients, and in **J.** patients≥ 60 years old, were dichotomized based on transition (trans) or no transition from T_EM_ to T_eff_ cells and LFS and OS were analyzed by the logrank test. A patient was considered transition-positive by the occurrence of a reduction of T_EM_ cells (%) and a simultaneous induction of T_eff_ cells (%) during the first treatment cycle.

When comparing the distribution of CD8^+^ T_N_, T_CM_, T_EM_ and T_eff_ cells before and after the first treatment cycle, non-relapsing patients showed a distinct reduction of the fraction of T_EM_ cells along with an induction of T_eff_ cells (Figure 2). At onset of therapy, patients with a high percentage (above the median) of T_EM_ cells showed a slightly higher likelihood of LFS, while no significant differences in relapse risk were found for patients with a high or low percentage (by the median) of T_N_, T_CM_, or T_eff_ cells (Figure S1). Patients experiencing a reduction of the frequency of T_EM_ cells or an induction of T_eff_ cells during cycle 1 showed significantly improved LFS and/or OS (Figure 3C-3D with results of multivariate analyses shown in Table 1). Also, induction of the frequency of naïve T cells during the first treatment cycle significantly predicted LFS and OS (Figure 3A, Table 1). The distribution of T_CM_ cells was seemingly unaltered during immunotherapy (Figure 2D) and did not influence relapse risk (Figure 3B). The distribution of T cell subsets was not significantly altered during treatment cycle 3 (data not shown).

**Table 1:** Univariate and multivariate Cox regression analyses of the impact of aspects of CD8^+^ T cell phenotype on LFS and OS

Cox regression analysis	Univariate			Multivariate*		
	HR	Conf. Interval	p-value	HR	Conf. Interval	p-value
**All patients (n=44)**						
Induction T_N_ vs LFS	0.45	0.20-0.98	0.04	0.47	0.21-1.06	0.07
Induction T_N_ vs OS	0.29	0.10-0.82	0.02	0.29	0.10-0.85	0.02
Induction T_CM_ vs LFS	1.08	0.50-2.36	0.84	1.46	0.64-3.32	0.37
Induction T_CM_ vs OS	1.43	0.52-3.93	0.49	2.65	0.91-7.76	0.08
Reduction T_EM_ vs LFS	0.26	0.12-0.60	0.001	0.26	0.11-0.60	0.002
Reduction T_EM_ vs OS	0.24	0.08-0.69	0.009	0.23	0.08-0.70	0.01
Induction T_EFF_ vs LFS	0.50	0.23-1.09	0.08	0.46	0.20-1.03	0.06
Induction T_EFF_ vs OS	0.35	0.12-0.99	0.048	0.27	0.09-0.80	0.02
Transition vs LFS **	0.19	0.07-0.50	0.001	0.17	0.06-0.47	0.001
Transition vs OS **	0.13	0.03-0.59	0.008	0.12	0.02-0.54	0.006
**Patients ≥60yo (n=27)**						
Induction T_N_ vs LFS	0.25	0.88-0.68	0.007	0.20	0.07-0.61	0.005
Induction T_N_ vs OS	0.17	0.04-0.67	0.01	0.13	0.03-0.59	0.008
Induction T_CM_ vs LFS	1.39	0.50-3.85	0.53	1.41	0.50-3.97	0.52
Induction T_CM_ vs OS	3.37	1.01-11.2	0.048	3.77	1.08-13.2	0.04
Reduction T_EM_ vs LFS	0.10	0.03-0.38	0.001	0.10	0.03-0.39	0.001
Reduction T_EM_ vs OS	0.14	0.03-0.57	0.006	0.13	0.03-0.57	0.006
Induction T_EFF_ vs LFS	0.22	0.07-0.68	0.009	0.26	0.08-0.87	0.03
Induction T_EFF_ vs OS	0.10	0.03-0.40	0.001	0.10	0.02-0.51	0.005
Transition vs LFS **	<0.001	***		<0.001	***	
Transition vs OS **	<0.001	***		<0.001	***	

Abbreviations: LFS, leukemia-free survival, OS, overall survival

* Adjusted for age and number of induction cycles

** Transition: reduction of T_EM_ with simultaneous induction of T_eff_

*** To few events for Cox regression analysis

Stimulation of CD8^+^ T cells with antigen has been proposed to drive differentiation from T_N_→T_CM_→T_EM_→T_eff_ cells [14, 15]. We thus speculated that T_eff_ cells were induced from T_EM_ cells during the first treatment cycle of HDC/IL-2. Indeed, a correlation was observed between an induction of T_eff_ and a reduction of T_EM_ during cycle 1 (Figure S2). Eighteen out of 44 analyzable patients (41 %) showed a memory to effector T cell transition, defined as a reduction of CD8^+^ T_EM_ cells with a concomitant increase in CD8^+^ T_eff_ cells. These patients showed improved LFS (HR 0.19, p < 0.001) and OS (HR 0.13, p = 0.002; Figure 3E, Table 1).

### T cell phenotypes in older patients

The trial protocol specified analyses of outcome by subgroups according to patient age at enrollment ( < 60 and > 60 years). The CD8^+^ T cell count or the distribution of CD8^+^ T_N_/T_CM_/T_EM_/T_eff_ cells at onset of immunotherapy did not differ significantly between age groups (Figure S3A-S3D). During the first cycle, treatment with HDC/IL-2 induced a significant increase of the frequency of T_eff_ cells only in older patients (Figure S3D). The impact of the induction of T_N_ and T_eff_ cells, the reduction of the frequency of T_EM_ cells and the apparent transition of T_EM_ cells into T_eff_ cells on outcome was pronounced in older patients. All of these aspects of immunotherapy-induced CD8^+^ T cell differentiation thus heralded LFS and/or OS in this age group (Figure 3F-3J) and remained significantly predictive after correction for prognostic factors (Table 1). The dynamics of CD8^+^ T cell subsets during cycle 1 did not significantly prognosticate LFS or OS in younger patients (Figure S3E-S3N).

### Additional markers of T cell activation

**Figure 4:**
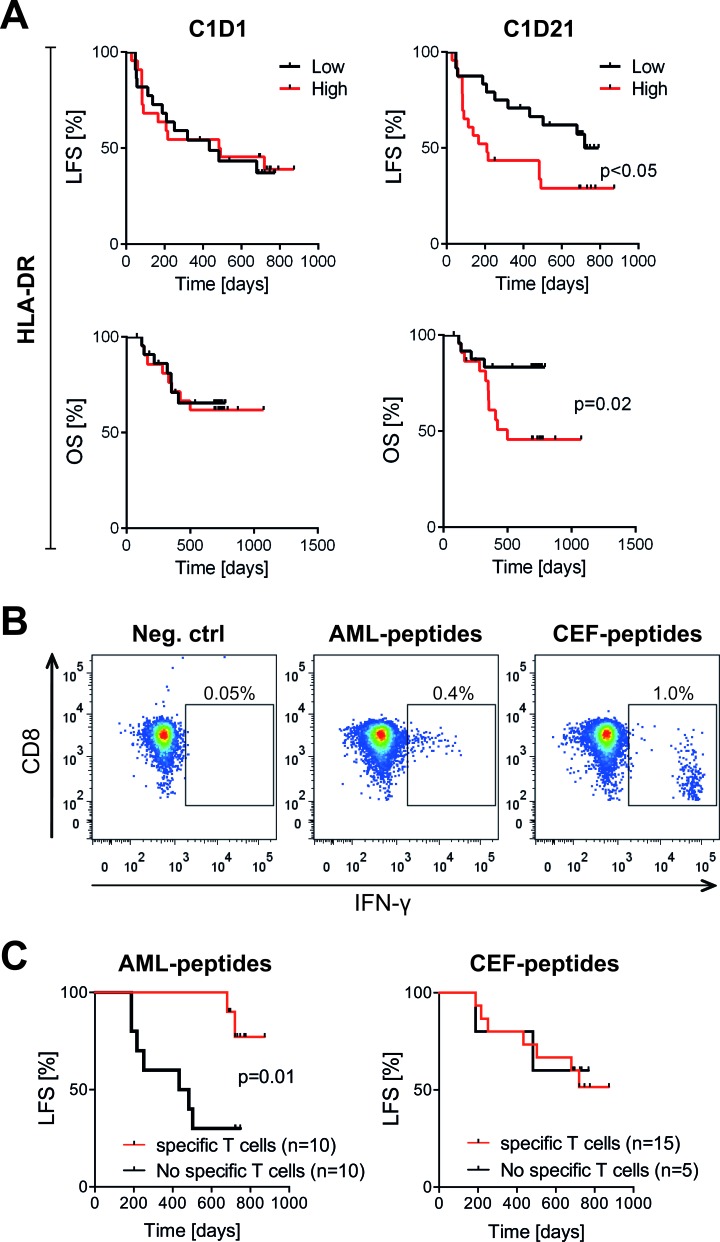
Impact of HLA-DR expression and leukemia-specific CD8^+^T cells on LFS in patients receiving HDC/IL-2 **A.** Patients were dichotomized by the median HLA-DR expression on CD3^+^CD8^+^ T cells at onset of therapy (C1D1; *n* = 44) or after the first treatment cycle (C1D21; *n* = 47). LFS and OS were analyzed by the logrank test. **B**-**C.** Blood samples from patients undergoing HDC/IL-2 treatment were stimulated with a pool of peptides from leukemia-associated antigens (AML-peptides) or a pool of peptides from CMV, EBV and influenza viruses (CEF-peptides), or no peptides (negative control). The percentage of IFN-γ producing CD8^+^ T cells was determined by flow cytometry. In **B.** representative dot plots show IFN-γ production in samples without stimulation and samples stimulated with AML- or CEF-peptides. In **C.** patients were dichotomized based on the presence or absence of AML-specific or CEF-specific CD8^+^ T cells, followed by analysis of LFS by the logrank test. Only patients with no events occurring before the last time point of analysis of antigen-specific T cells (C3D21; 105 days) were considered in the latter analyses.

In addition to the distribution of T cell phenotypes we analyzed the impact of the immunotherapy on cytotoxic T cell activation markers. The expression levels of CD69 and CD25, which are frequently employed markers of activated T cells, were unaffected by HDC/IL-2 treatment (Figure S4A-S4B) and the expression levels of these markers did not influence clinical outcome (data not shown). In contrast, the expression of HLA-DR was reduced during the first cycle of therapy (Figure S4C). HLA-DR was mainly expressed by the memory populations, and the reduction was significant for T_CM_ and T_EM_ cells but not T_N_ or T_eff_ cells (data not shown). A low expression of HLA-DR on CD8^+^ T cells at the end of the first HDC/IL-2 treatment cycle weakly predicted a favorable clinical outcome (univariate Cox regression analysis for OS was p = 0.04, while multivariate Cox regression analysis for OS was p = 0.12; Figure 4A).

### Presence of leukemia-specific T cells heralds maintained CR

We next determined the ability of CD8^+^ T cells to produce IFN-ɣ *ex vivo* before and after immunotherapy. The capacity of patients' CD8^+^ T cells to produce IFN-ɣ after stimulation with PMA/ionomycin was similar before and after the first treatment cycle (Figure S4D) and did not impact on the clinical outcome (not shown). To determine whether patients harbored CD8^+^ T cells that were specifically reactive with leukemic antigens, PBMCs were stimulated by peptide pools representing known leukemia-associated antigens (WT1, survivin, PRAME and hTERT) followed by quantification of IFN-γ-producing CD8^+^ T cells. Healthy donor CD8^+^ T cells from PBMCs did not produce above background levels of IFN-γ in response to the leukemia-derived peptides (data not shown). Three out of 20 analyzed patients displayed antigen-specific CD8^+^ T cells against any of these antigens at onset of immunotherapy (C1D1). Two of these patients experienced late relapses (at > 600 days). Seven patients acquired leukemia-reactive T cells during immunotherapy (at C1D21, *n* = 2, C3D1, *n* = 4 or C3D21, *n* = 1), all of whom remained in uninterrupted CR. By Kaplan-Meier analysis, presence of leukemia-specific CD8^+^ T cells predicted LFS (p = 0.01) whereas presence of antigen-specific CD8^+^ T cells responding to viral control peptides (CMV, EBV and influenza; CEF) did not (p = 0.5; Figure 4B-4C).

## DISCUSSION

The results of this study imply, for the first time, that an altered distribution of cytotoxic T cell phenotypes in blood during immunotherapy may be relevant to the prognosis of non-transplanted AML patients in CR. A major finding was that these aspects of T cell immunity determined the relapse risk and survival of older patients, who are at high risk of relapse and death [16]. Our results also point to conceivable biomarkers for efficacy, including memory to effector T cell transition, which may be broadly useful in T cell-based cancer immunotherapy. The reason for the lack of significant correlation between the dynamics of CD8^+^ T cell subsets and outcome in younger patients is not known, but might be related to a lower incidence of relapse in this age group along with fewer samples available for analysis.

The precise mechanism explaining our finding of a shift from T_EM_ cells to T_eff_ cells in blood of AML patients during the first cycle of HDC/IL-2 immunotherapy remains to be determined. However, IL-2 has been reported to promote the development of CD8^+^ T cells into memory and effector cell populations (reviewed in [17]) and it is thus conceivable that the IL-2 component of the HDC/IL-2 regimen was crucial for the observed memory to effector T cell transition. Also, the memory to effector cell transition is compatible with the view that T_EM_ cells differentiate into T_eff_ cells after antigen exposure [14, 15]. While alternative explanations are possible, including extravasation of T cell subsets during immunotherapy, we hypothesize that immunotherapy with HDC/IL-2 facilitates the development of effector T cells, which may explain the strong prediction of clinical outcome in patients experiencing T_EM_ to T_eff_ transition. Of note, others have shown that AML-specific T cells carry a T_eff_ cell phenotype [18]. In further support for the development of functional T cell immunity during immunotherapy, detectable levels of CD8^+^ T cells that reacted with leukemic peptides evolved in 7/20 patients during the course of therapy. Presence of leukemia-specific CD8^+^ T cells, but not CD8^+^ T cells reactive with common viral antigens, significantly predicted LFS. These results concur with previous observations on a role for immunoreactive leukemia-associated antigens in AML [5, 6, 19] and lend support to the role of cytotoxic T cells for surveillance of the leukemic clone.

HLA-DR is considered a T cell activation marker [20], but increased expression of HLA-DR on CD8^+^ T cells has also been linked to T cell suppression and exhaustion in cancer, chronic virus infections and aging [21, 22]. The reduction of HLA-DR expression in T_CM_ and T_EM_ cells during immunotherapy and the trend towards favorable clinical outcome among patients with reduced expression suggest that the memory population of CD8^+^ T cells may be shifted towards improved effector function during immunotherapy, but further studies are required to confirm this hypothesis.

**Figure 5:**
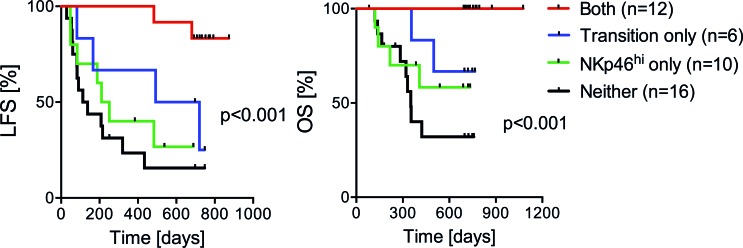
Impact of T_EM_ to T_eff_ cell transition and NK cell NKp46 expression on clinical outcome **A.**-**B.** Patients were regarded as transition-positive when showing a reduction of T_EM_ cells (%) and a simultaneous induction of T_eff_ cells (%) during the first treatment cycle, and were considered NKp46^high^ when their CD16^+^ NK cells expressed above median levels of NKp46 after the first cycle of immunotherapy (C1D21). Data show the LFS and OS (analyzed by the logrank test for trend) of patients with transition T_EM_-T_eff_ and NKp46^high^ (both), transition only, NKp46^high^ only, no transition T_EM_-T_eff_ and low NKp46 expression (neither).

HDC/IL-2 has been developed for AML immunotherapy to expand and activate populations of T cells and natural killer (NK) cells (IL-2 component) and concurrently protect these anti-leukemic effector cells against inactivation by myeloid cell-derived reactive oxygen species (HDC component) [23]. Earlier results from the Re:Mission trial show that treatment with HDC/IL-2 triggered expression of natural cytotoxicity receptors (NCR), which are activating receptors of importance for NK cell recognition of aberrant cells, along with increasing NK cell counts in blood [24, 25]. The Re:Mission trial results suggested that in-cycle increments of NCR expression and NK cell counts only weakly predicted LFS and OS [24], whereas the results presented herein imply that altered distribution of CD8^+^ T cell subsets during the first treatment cycle was critical to prognosis. Additional studies are required to define the relative contribution by NK cells and T cells in mediating the anti-leukemic properties of HDC/IL-2 and in surveillance of the leukemic clone in AML. We observed that patients with a T_EM_ to T_eff_ cell transition during the first treatment cycle along with high NKp46 expression on cytotoxic CD16^+^ NK cells after immunotherapy were strikingly protected from relapse, while patients with T_EM_ to T_eff_ cell transition or high NKp46 expression alone were only partly protected (Figure 5). These data support the hypothesis that T and NK cell effector functions evolve simultaneously during immunotherapy and that both cell subsets may contribute in relapse protection.

A limitation to this study is that relatively few patients were available for assessment of T cell phenotype and function, and larger studies are required to confirm our observations. Also, the observed dynamics of CD8^+^ T cell subsets during treatment with HDC/IL-2 may reflect an inherent survival advantage of a subgroup of patients with functional cellular immunity rather than effects of immunotherapy. Randomized comparisons, preferably within the group of older patients at high risk for relapse, should be performed to further define the role of CD8^+^ T cell dynamics for the course of AML and in AML immunotherapy.

## PATIENTS AND METHODS

### Patients, study design and objectives

This single-armed multicenter phase IV study (Re:Mission, NCT01347996, registered at www.clinicaltrials.gov) enrolled 84 patients (age 18-79) with AML in first CR. As outlined schematically in Figure 1, the patients received ten consecutive 21-day cycles of histamine dihydrochloride (HDC; Ceplene) in combination with low-dose IL-2 during 18 months or until relapse or death. Patients were monitored for at least six additional months after the end of immunotherapy. Patients who discontinuated prematurely from the study (9 due to adverse events, 3 due to withdrawn concent and 2 for other reasons) were sensored at the last captured follow-up date The dosage, route of administration, exclusion criteria etc. were identical to those described for a previous phase III trial [10]. Primary endpoints in the Re:Mission study included assessment of the quantitative and qualitative pharmacodynamic effects of HDC/IL-2 by monitoring T and NK cell phenotypes before and after treatment cycles. The protocol stated that all data collected in support of these objectives were to be analyzed for the populations as a whole and by subgroups according to patient age at enrolment ( < 60 and > 60 years). The herein reported aspects of T cell biology *vs*. clinical outcome (LFS and OS) were performed post-hoc. Patient characteristics are presented in Table 2. A more detailed account for previous induction and consolidation therapy can be found elsewhere [24, 25].

**Table 2:** Patient characteristics

	n (%)		LFS, n (%)	
	All patients (n=84)	Age ≥60 (n=47)	All patients	Age ≥60
**Sex**				
Female	44 (52)	23 (49)	15/44 (34)	6/23 (26)
Male	40 (48)	24 (51)	20/40 (50)	10/24 (42)
**Risk group**				
Favorable risk	34 (40)	17 (36)	18/34 (53)	8 /17 (47)
Intermediate I	25 (30)	10 (21)	9/25 (36)	2/10 (20)
Intermediate II	13 (15)	9 (19)	6/13 (46)	4/9 (44)
High risk	7 (8)	6 (13)	1/7 (14)	1/6 (17)
ND	5 (6)	5 (11)	1/5 (20)	1/5 (20)
**Karyotype**				
Normal	44 (52)	23 (49)	19/44 (43)	9/23 (39)
Favorable	14 (17)	5 (11)	8/14 (57)	2/5 (40)
Unfavorable	7 (8)	6 (13)	2/7 (29)	2/6 (33)
Other	15 (18)	10 (21)	5/15 (33)	3/10 (30)
ND	4 (5)	3 (6)	1/4 (25)	0/3 (0)
**Mutation status**				
NPM1	**n=69** 25 (36)	**n=39** 14 (36)	12/25 (48)	6/14 (43)
FLT3	**n=72** 6 (8)	**n=37** 3 (8)	0/6 (0)	0/3 (0)
CEBPA	**n=42** 3 (7)	**n=23** 1 (4)	1/3 (33)	0/1 (0)
**Induction courses**				
1	63 (75)	33 (70)	31/63 (49)	14/33 (44)
>1	21 (25)	14 (30)	4/21 (19)	2/14 (14)
**Consolidation courses**				
0-2	41 (49)	27 (57)	15/41 (37)	6/27 (22)
>2	43 (51)	20 (43)	20/43 (47)	10/20 (50)

Abbreviations: LFS, leukemia-free survival, ND, not done

### Sampling of peripheral blood and flow cytometry

Peripheral blood was collected before and after the first and third treatment cycles, i.e. cycle 1, day 1 (C1D1) and cycle 1, day 21 (C1D21), cycle 3, day 1 (C3D1) and cycle 3, day 21 (C3D21). PBMC were isolated and cryopreserved at local sites and shipped on dry ice to the central laboratory (at the Sahlgrenska Cancer Center, University of Gothenburg, Sweden) for analysis by use of flow cytometry. For these analyses, the frozen PBMC samples were thawed quickly in Iscoves' medium supplemented with 10% FCS. Subsequently, cells were washed in Iscoves' medium and thereafter in PBS. Cells were first stained with LIVE/DEAD fixable yellow stain (Life technologies, Grand Island, NY, USA) by incubation for 30 min at 4°C in PBS, followed by staining for surface markers for 30 min at 4°C in PBS containing 0.5% BSA and 0.1% EDTA or in brilliant stain buffer (BD Biosciences, Stockholm, Sweden). The following anti-human monoclonal antibodies were used for phenotyping: CD3-FITC (HIT3a), CD4-APC-H7 (RPA-T4), CD4-Horizon V450 (RPA-T4), CD8-APC (RPA-T8), CD8-PerCP-Cy5.5 (RPA-T8/SK1), CD8-Qdot705 (3B5), CD16-Horizon V450 (3G8), CD25-Brilliant Violet 421 (M-A251), CD45RA-APC (HI100), CD45RO-PE (UCHL1), CD56-PerCP-eFluor710 (CMSSB), CD56-PE-Cy7 (NCAM16.2), CD69-PE-Cy7 (FN50), HLA-DR-FITC (L243) (all from BD Biosciences). CCR7-PE-Cy7 (G043H7) from Biolegend, San Diego, CA, USA. CD3-Pacific Blue (S4.1), CD14-Qdot655 (TüK4) and streptavidin-Qdot605 (all from Life technologies). Intracellular staining of IFN-ɣ-PE-Cy7 (B27; BD Biosciences) was performed after surface staining and fixation and permeabilization using the FoxP3 fixation/permeabilization kit (eBioscience, San Diego, CA, USA) according to the manufacturer's instructions.

Stained samples were analyzed on a 4-laser BD LSRFortessa SORP flow cytometer (405, 488, 532, and 640 nm; BD Biosciences). Data were analyzed using the FlowJo software, version 7.6.5 or later (TreeStar, Ashland, OR, USA) or the FACSDiva software, version 6 or later (BD Biosciences). Samples with less than 25 % viability were excluded.

Blood samples were available from 81 out of 84 patients. Differential counts of whole blood were performed at local sites and were utilized to calculate absolute counts of blood CD8^+^ T cells. All available samples were analyzed for T cell content and expression of activation markers (including CD25 and CD69 and IFN-γ production in response to PMA). If an analysis failed according to pre-defined criteria (experimental failure, few cells, poor cellular viability), a second sample was thawed for re-analysis. If also the second attempt failed to generate data, these samples were excluded from analysis. In a second set of experiments, available samples were analyzed for distribution of T cell subsets and HLA-DR expression. All successfully analyzed samples, according to the pre-defined criteria stated above, were included in this report. A flow chart of patients that were included or excluded from the analyses is shown in Figure S5.

### IFN-ɣ-production after PMA/ionomycin or peptide stimulation

Thawed samples collected before and after the first and third treatment cycles of HDC/IL-2 immunotherapy were seeded in Iscoves' medium supplemented with 10 % FCS in 96-well plates, 1×10^6^ cells per well. Cells were left to rest over night at 37°C. The next day, cells were washed with warm medium before stimulation consisting either of a 5 hours incubation with 0.2 μg/ml PMA (Sigma-Aldrich Munich, Germany) together with 2 μg/ml ionomycin (Sigma-Aldrich) or a 6 hours incubation with a pool of AML-peptides (overlapping peptides covering the leukemia-associated proteins WT1, PRAME, survivin and hTERT; Miltenyi Biotec) or, as a control, CEF-peptides (32 peptides specific for MHC class I with sequences derived from human cytomegalovirus (HCMV), Epstein-Barr virus (EBV) and influenza viruses; Miltenyi Biotec).

The final concentration of each peptide was 0.6 nmol. The co-stimulatory molecules anti-CD28 (CD28.2; BD Biosciences) and anti-CD49d (9F10; BD Biosciences), 2 μg/ml of each, were added to wells with peptide stimulation, as well as to the negative control samples, which served to determine the background signal. For all stimuli, Golgiplug (BD Biosciences) was added during the last 4 hours of stimulation according to manufacturer's protocol. Samples were stained with LIVE/DEAD fixable yellow stain and surface markers before being fixed, permeabilized and intracellularly stained with IFN-ɣ as described above. Patients were considered to harbor AML- or CEF-specific CD8^+^ T cells if the frequency of IFN-ɣ-producing CD8^+^ T cells exceeded 0.05 % (after subtraction of background as determined by the negative control sample) at any of the time points (C1D1, C1D21, C3D1, C3D21). To avoid selection bias, only patients with no events (relapses) occurring before the last time point (C3D21; 105 days) were considered in these analyses.

### Statistics

In accordance with the statistical analysis plan, paired *t*-test was used for single comparisons of CD8^+^ T cell phenotypes. To determine the impact of the dynamics of CD8^+^ subsets or markers on outcome, samples were dichotomized by the median for single time points or by induction/reduction during the first treatment cycle if not otherwise stated. The analyses of T cell function *vs*. outcome are based on data for LFS, defined as the time in days from start of immunotherapy with HDC/IL-2 to relapse or death from any cause, and OS, defined as the corresponding time to death, available at the trial closing date (October 13, 2014), i.e. when all patients had been followed for at least 24 months (18 months of treatment and 6 months of additional follow-up). Relapse was defined as at least 5% blast cells in the bone marrow or presence of extramedullary leukemia. The impact of T cell phenotype on LFS and OS was analyzed using the log-rank test.

The impact of age, risk group classification according to recommendations by the European LeukemiaNet [26], number of induction courses required to achieve CR (1 or > 1), and number of consolidation courses (0-2 or > 2) on LFS and OS was assessed using the Cox univariate regression model. The prognostic factors with a p-value below 0.1 (age, and number of induction cycles) were included as potential confounders in a Cox multivariate regression analysis (Table 1). All indicated p-values are 2-sided. This study was conducted according to Declaration of Helsinki principles. The trial was approved by the Ethics Committees of each participating institution, and all patients gave written informed consent before enrollment.

## SUPPLEMENTARY MATERIAL FIGURES


