# Supplementary material for: Dynamics of cytotoxic T cell subsets during immunotherapy predicts outcome in acute myeloid leukemia
Source: Oncotarget. 2016 Feb 5;7(7):7586–96. doi: 10.18632/oncotarget.7210 (PMC4884940; doi:10.18632/oncotarget.7210)
Supplement: Supplementary file 1 [file oncotarget-07-7586-s001.pdf]

Dynamics of cytotoxic T cell subsets during immunotherapy predicts outcome in acute myeloid leukemia

Supplementary Material

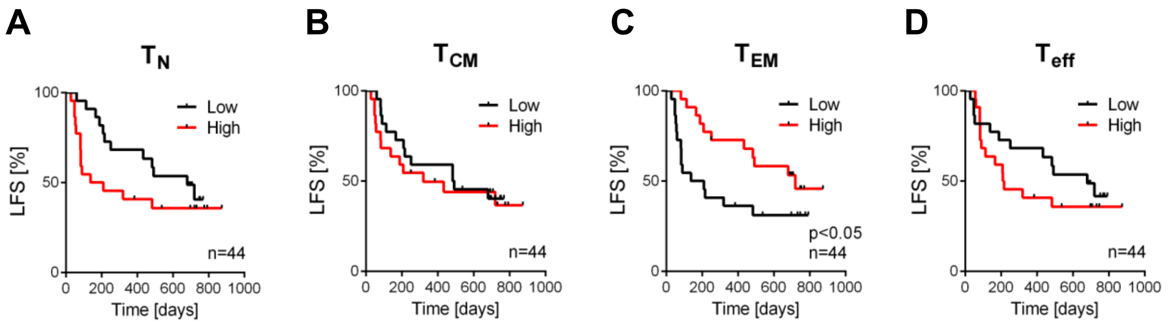

Supplementary figure 1. Impact of CD8$^+$ subsets at the onset of immunotherapy on LFS. Patients were dichotomized based on the median percentage of T_N **(A)** T_{CM} **(B)**, T_{EM} **(C)** and T_{eff} **(D)** CD8$^+$ T cells in blood samples collected before the first treatment cycle of HDC/IL-2. LFS was analyzed by the logrank test.

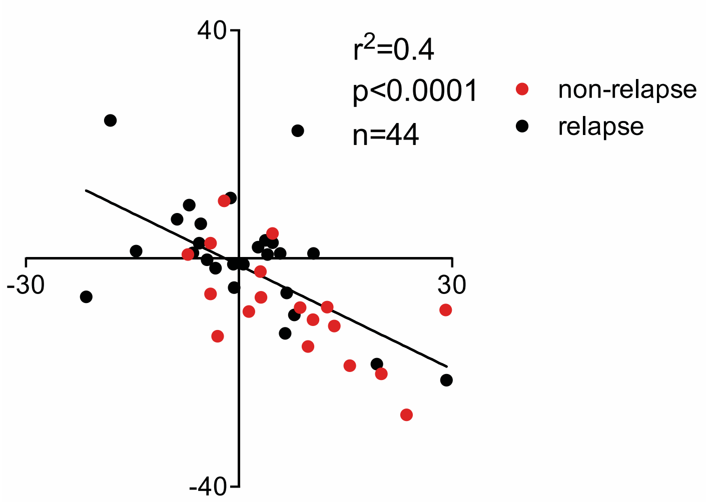

Supplementary figure 2. Negative correlation between changes in the distribution of T_{EM} and T_{eff} cells during immunotherapy. Changes in the frequency of $CD8^{+}$ T_{EM} (y-axis) and T_{eff} (x-axis) during the first treatment cycle with HDC/IL-2 were correlated. Non-relapsing patients are marked by red dots, while relapsing patients are marked by black dots.

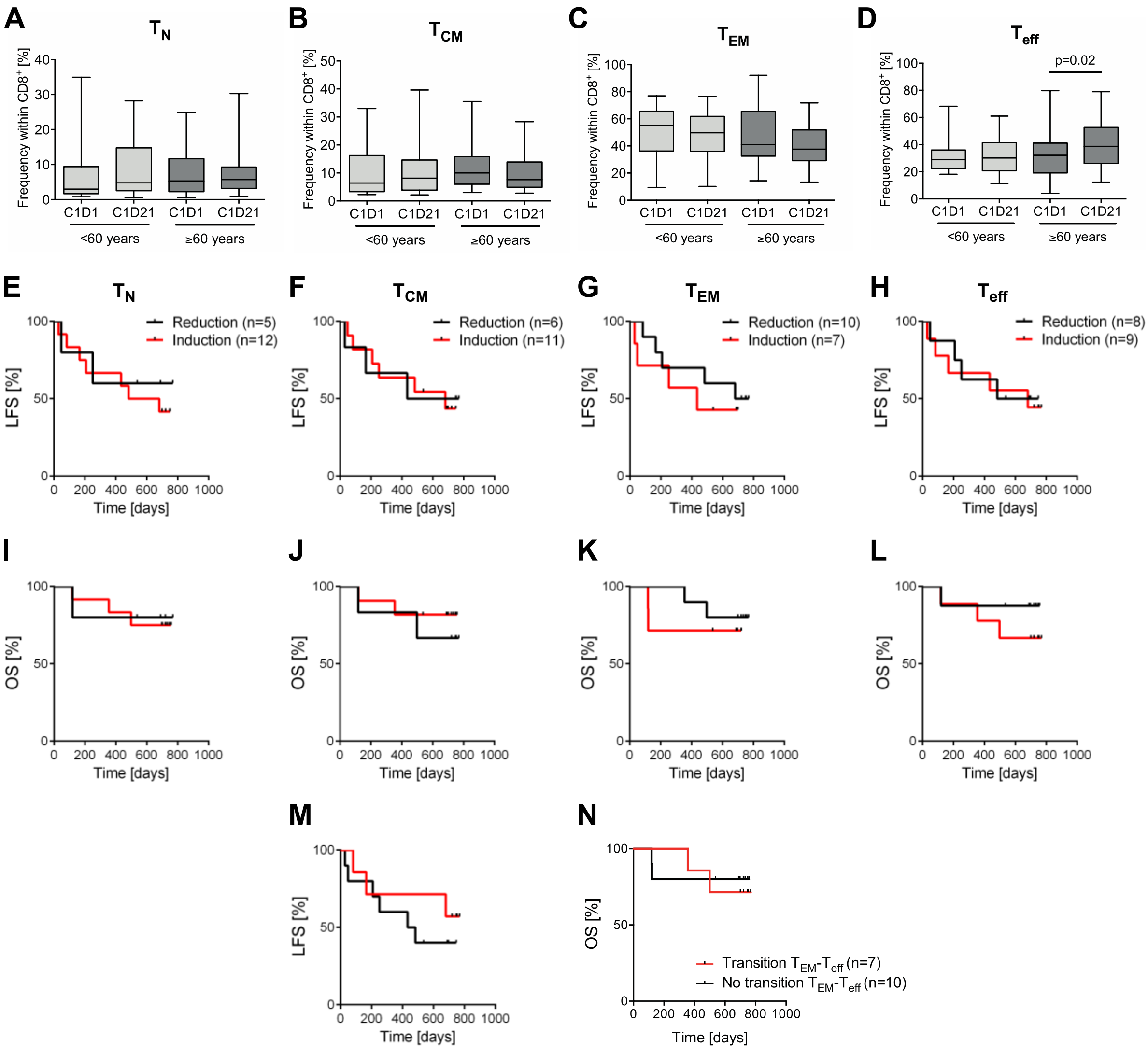

Supplementary figure 3. Distribution of CD8$^+$ T cell populations in younger and older patients receiving HDC/IL-2. (A-D) Frequency of the CD8$^+$ subpopulations T_N, T_{CM}, T_{EM} and T_{eff} cells in patients <60 years old (n=17) and patients >60 years old (n=27) before (cycle 1, day 1; C1D1) or after (C1D21) cycle 1 of HDC/IL-2 immunotherapy. Statistical analysis was performed by Student's paired t-test. In (**E-L**) patients <60 years old were dichotomized based on induction or reduction of the frequency of the different CD8$^+$ subsets during the first treatment cycle, followed by analyses of LFS and OS by the logrank test. In (**M-N**), patients <60 years old were dichotomized based on transition or no transition from T_{EM} to T_{eff} cells and LFS and OS were analysed by the logrank test.

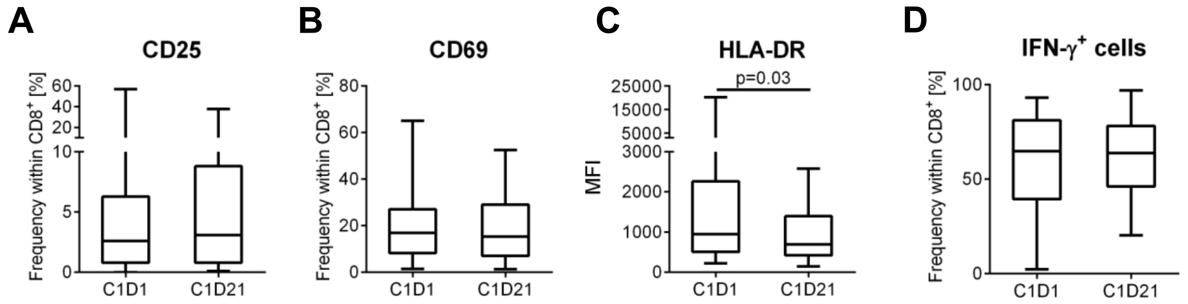

Supplementary figure 4. Expression of activation markers on CD8$^+$ T cells during immunotherapy with HDC/IL-2. Frequency of (**A**) CD25$^+$ and (**B**) CD69$^+$ cells within the CD3$^+$CD8$^+$ population (cycle 1, day 1; C1D1 n=62; C1D21 n=63). **(C)** Median fluorescence intensity (MFI) of HLA-DR on CD3$^+$CD8$^+$ T cells (n=44). **(D)** Blood samples collected at the onset (C1D1) or end of (C1D21) the first cycle of immunotherapy were stimulated with PMA/ionomycin followed by intracellular staining of IFN-γ. The box plots show the frequency of IFN-γ-producing CD8$^+$ T cells before and after the first treatment cycle (C1D1 n=58; C1D21 n=63). Statistical analysis was performed by Student's paired t-test. The reduction of HLA-DR intensity during cycle 1 (shown in panel **C**) remained statistically significant in non-parametric analysis (Wilcoxon signed-rank test, p=0.0003).

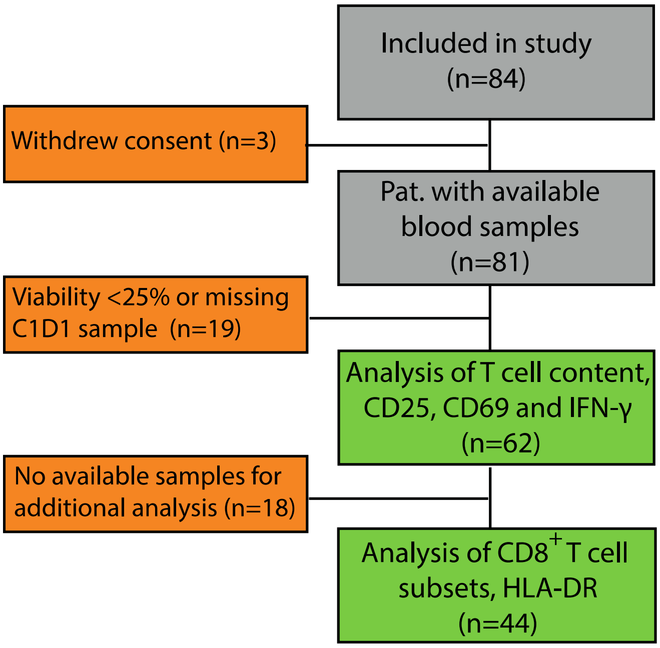

Supplementary figure 5. Analysed patients. Flow chart showing the number of patients included to the study and the number of successfully analysed samples.
